# Minimizing the Energy Hole Problem in Wireless Sensor Networks: A Wedge Merging Approach †

**DOI:** 10.3390/s20010277

**Published:** 2020-01-03

**Authors:** Nusrat Sharmin, Amit Karmaker, William Luke Lambert, Mohammad Shah Alam, MST Shamim Ara Shawkat

**Affiliations:** 1 Institute of Information and Communication Technology, Bangladesh University of Engineering and Technology, Dhaka 1000, Bangladesh; nusrat.sharminbd@gmail.com (N.S.); amitkarmaker06@gmail.com (A.K.); 2 Department of Computer Science, Tennessee Technological University, Cookeville, TN 38505, USA; wllambert42@students.tntech.edu; 3 Department of Electrical Engineering and Computer Science, University of Tennessee Knoxville, Knoxville, TN 37996, USA

**Keywords:** energy hole, clustering, chain formation, wireless sensor network

## Abstract

The Energy hole problem, a common phenomenon in wireless sensor networks, significantly decreases the lifetime of any deployed network. Some of the popular techniques to minimize such problems are using mobile sinks instead of static sinks, extending the transmission range dynamically, and deploying redundant sensor nodes near the base station/sink. The major drawback to these techniques are that energy holes may still be created at some point due to their static nature of deployment, despite having the overall residual energy very high. In this research work, we adopt a new approach by dividing the whole network into equiangular wedges and merging a wedge with its neighboring wedge dynamically whenever individual residual energy of all member nodes of a wedge fall below a threshold value. We also propose an efficient Head Node (HN) selection scheme to reduce the transmission energy needed for forwarding data packets among Head Nodes. Simulation results show that WEMER, our proposed WEdge MERging based scheme, provides significantly higher lifetime and better energy efficiency compared to state-of-the-art Power-Efficient Gathering in Sensor Information Systems (PEGASIS) and contemporary Concentric Clustering Scheme (CCS), and Multilayer Cluster Designing Algorithm (MCDA).

## 1. Introduction

Wireless Sensor Networks (WSNs), a collection of a large number of resource constraint sensor nodes [1], are widely used in applications like environment monitoring, military surveillance, disaster forecasting, agriculture, remote patient monitoring, factory automation [2,3,4], etc. In such applications, the main task of the sensor nodes is to collect the relevant data from the environment and send this gathered data to the Base Station (BS) or sink through multi-hop communication. Nodes closer to the sink or BS in multi-hop WSNs need to transmit or forward more traffic than other distant nodes of the network. This topological disadvantage makes the closer nodes drain out their energy at a significantly higher rate, which may create energy holes in the network [5].

**Figure 1:**
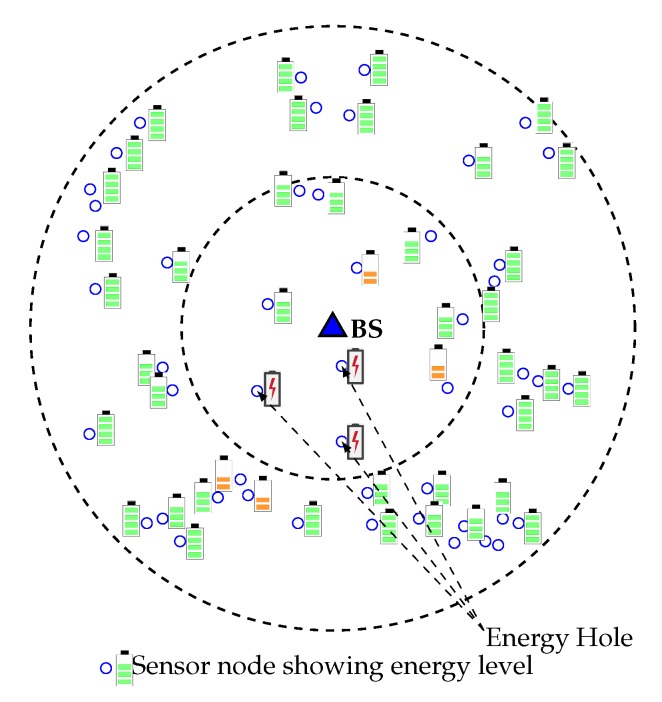
Energy hole problem in Wireless Sensor Networks (WSNs).

As a result, outer layer sensor nodes cannot forward data to the sink, which eventually decreases the network lifetime, even in case of high overall residual energy. Figure 1 gives an overview of the energy hole situation in which nodes closer to the Base Station have low remaining battery power, and nodes that are far away from BS have high remaining battery power. The energy hole problem can also happen if any nodes in the network receive and/or transmit more packets than the other nodes [6]. In a multi-hop network scenario, the overall network can collapse due to this energy hole problem. So, balancing the energy consumption among the deployed nodes is therefore of great importance, and grouping the nodes dynamically and more efficiently is an up-and-coming technique to achieve such a goal. A lot of medium access protocols are introduced in the last decade for improving the energy efficiency of sensor networks, but few of them consider the adverse effect of energy hole [7]. In this paper, we propose a new WEMER mechanism based on WEdge MERging to improve the energy efficiency of the network considering the negative impact of the energy hole. The major contributions of this paper are listed below:

- Unlike other existing protocols, the proposed WEMER protocol considers the distance between the HN of the immediate inner corona and the prospective HNs of the current corona while selecting an HN. Since HN of each corona forwards messages that it accumulates from its member nodes to the HN of the inner corona, a prospective HN may not be the optimal one unless its distance to the HN of the inner corona is considered, especially in case of multi-hop communication. WEMER, therefore, starts HN selection from the innermost corona so that the nodes from the successor corona can calculate their distance from the predecessor HN. While most of the other HN selection algorithms consider the distance between a prospective HN and its member nodes as the main selection factor, this paper also considers the distance between a prospective HN and its predecessor. By utilizing this distance as well as residual energy information, a distributed advertisement mechanism selects the most efficient HN for a sector.
- We also improve the reconstruction procedure of the network by introducing a dynamic wedge merging approach whenever an energy hole situation occurs. This distributed approach checks Residual Energy (RE) of each sector in every round, and if the RE of a sector is less than a predefined threshold, it merges with one of its neighbor sectors that has higher RE.
- Finally, we also improve the chain construction of a sector to avoid long link problem. Whenever a distance threshold is reached while forming a chain within a sector, it selects more than one HN to reduce the delay associated with long-distance communication.

A very preliminary version of this research work has been presented in [8]. The remainder of this paper is organized as follows. In Section 2, we describe the preliminaries related to all concepts and provide a summary of state of the art. Our proposed work is described in detail in Section 3. Section 4 is dedicated to results and performance analysis. Finally, we conclude the paper in Section 5.

## 2. Related Work

Nodes deployment is the first step in establishing a sensor network. Sensor nodes are battery powered and randomly deployed in the target area. The significant challenges of the sensor nodes are battery power limitations, processing power constraints, duplicate data gathering, and the limited memory of the network. Optimizing energy consumption is one of the significant tasks in WSNs, in order to prolong the network lifetime. For addressing the issue, researchers focused more on this area during the last few years. If the sensor nodes are deployed uniformly, nodes near the sink send their own data, as well as the data collected by other nodes, away from the sink, in a multi-hop scenario. In this case, the sensor nodes that are nearest to the sink consume more energy and die out quickly [9,10,11]. On the other hand, if multi-hop is not used and all nodes transmit their data directly to the BS, the nodes farthest from the BS die much more quickly than the nodes that are closer to the BS, this is because they need more transmission power to transmit their data to the BS [12]. As a result, the sensor network is disconnected, having sufficient energy left unused [13], which causes a significant decrease in the network lifetime. Energy holes create a partition in the network in such a way that it cannot make full connectivity in the network. Various techniques have been proposed to address the Energy Hole Problem (EHP) [14]. Some techniques that alleviate the energy hole problem include: adjustable transmission range [15], compressed sensing [16], sink or node mobility, and non-uniform sensor distribution, which mainly deploys redundant nodes near the sink [17]. The aim of an energy hole avoidance is to delay or bypass the formation of the energy hole to maximize the network lifetime.

Based on the network structure Pantazis et al. [18] categorized the routing protocol of WSN into flat and hierarchical routing. In large scale network, hierarchical protocol improves energy efficiency than flat routing. The hierarchical protocol is further divided into clusters, chain, tree, grid, etc. Clustering is an efficient way to both save energy and prolong the network lifetime [19,20,21,22,23]. For large scale networks, clustering is a popular approach. In this approach, every member of the cluster sends their data to the cluster head, in order to transmit it to the base station. Based on the residual energy of the node and the average energy of the cluster, the Adaptive Decentralized Re-clustering Protocol (ADRP) chooses the set of the next cluster heads for upcoming rounds [24]. But until the next initial phase, no new node can be added, and if any next head node dies, the cluster will not be formed. In [25] the authors restrict the number of CH advertisement to the optimal number of the CH count during CH selection time, in order to save energy. A lot of clustering protocol has been proposed in various literature but they doesnot consider the energy hole situation [26,27,28,29,30,31].

Several mobile sensor-based protocols are proposed for achieving the energy efficiency of WSNs [32] Yarinezhad et al. [33] proposed a cluster-based protocol based on virtual grid infrastructure and mobile sink to fairly utilizing the energy consumption of sensor nodes by reducing traffic load in the network. They partitioned the sensing area into several same dimensioned region. Some nodes that are near the intersecting region of the virtual grid are responsible for tracking the recent position of the mobile sink. The sensing node sends its data to the mobile sync by using a geographic routing algorithm through the location information gathered from the intersecting region node. The key feature of this protocol is to minimize the communication cost between the mobile sink and sensing nodes. But, it increases the traffic load of the responsible node. Toor et al. [34] proposed Multi-hop Energy Efficient Routing Protocol using Multiple Mobile Nodes (MEACBM) protocol for WSN. They propose a new probabilistic CH selection algorithm and form several clusters and sub-clusters. Then, the sensing region is divided into several sectors and each sector contains multiple clusters and sub-clusters. A mobile data collector (MDC) is placed in each sector to collect data from the CH. It reduces the transmission cost of CH. However, it requires proper synchronization among the CHs and MDC. Besides, MDC consumes more energy due to the high traffic load.

On the other hand, PEGASIS [35] used greedy algorithm to construct a chain. Chain construction procedure is started from the farthest node from the base station. However PEGASIS has some limitations, firstly, One chain is form for whole network which creates long chain and long chain makes delay of communication. Secondly, only leader node can transmit data to the base station. So leader node is heavily loaded. Thirdly, when PEGASIS protocol selects leader node, energy or distance is not considered. Fourthly, it is essential to have a complete view of the topology at each and every node for chain construction. Finally, greedy algorithm create long link problem in large network, that makes energy consumption of node is high. Another Chain Based Cluster Cooperative Protocol (C BCCP) [36] partitions the network into some subareas (clusters). Each subarea contains CH and Cluster Coordinator (CCO) to distribute the load defined number of the relay node. The number of CCO depends on the position of the cluster, since there is one CCO for every below lying cluster of it. CBCCP randomly select CH, CCO, and do not consider the RE or distance so, there is a probability that a node with low RE can be selected as CH or CCO instead of a high RE node.

CCS [37] is an enhanced of PEGASIS protocol. The main improvement of CCS is that it consider base station position when divide the network into concentric circles. The term concentric clustering means that the shape of a cluster is concentric circles when cluster is formed. CCS improves the delay problem of PEGASIS but there is unbalanced node distribution at each level, which will cause the levels with a small number of nodes to deplete their energy first. Furthermore, during head node selection of each level, energy and distance are not considered which creates unbalanced energy consumption.

Multilayer cluster designing algorithm (MCDA) [38] for improving energy efficiency is a combination of Distributed Cluster Designing (DCD) and Centralized Cluster Designing Approach (CCD). This protocol comprises of three steps: self organizing, flat layer design and clustered layer design. However, nodal density is the only metric to select a cluster head, so it makes the same node to select as cluster head again and again. Furthermore, number of cluster member for a cluster is not defined. That’s why cluster is not equally loaded. L. Prabha et al. [39] proposed an energy hole repelling algorithm by which sensor nodes closer to the BS creates smaller cluster and nodes far away from BS creates big cluster. Baniata et al. [40] proposed an Energy Efficient Unequal Chain Length Clustering (EEUCLC) protocol to overcome the energy hole problem, by creating an unequal chain within a cluster. However, there is no mechanism in the network face energy hole situation because cluster members near the BS still handle huge traffic loads from the successor clusters. Elkamel et al. [41] proposed unequal clustering in order to avoid the energy hole problem, by creating a smaller cluster near the BS and a large cluster far from the BS. This protocol distributes the energy load in the network, but does not ensure the mechanism if the energy hole problem exists. Wang et al. [42] proposed a layer based unequal clustering mechanism, using multi hop communication to avoid the energy hole problem. Zhao et al. [43] proposed an Immune Clone Selection-based Power Control (ICSPC) algorithm for solving the energy hole problem by using a cluster based coronal model. The dynamic CH selection, non-uniform node distribution, imbalance energy consumption in each corona and the transmission range adjustment is introduced to solve the energy hole problem. Reem et al. [44] has proposed On-hole children reconnection (OHCR) by considering local nature and on-hole alert (OHA) by considering global nature protocols to reconnect the network after the premature death of a node. In the case of a cluster or chain based protocol, the OHCR protocol increases the number of cluster head or chain head. The reason is whenever a parent node dies; its child nodes search for a new parent node within their ranges. If no other node (same level or upper level) within its range exist, it joins the BS directly. In chain based network, it forms another chain head. However, the number of chain head is closely related to the performance metrics. Too many chain head creates extra energy consumption in the network. Moreover, a significant number of control packets are also needed to exchange that increases energy consumption.

Naranjo et al. [45] proposed Prolong Stable Election Protocol (P-SEP) to prolong the network lifetime and to enhance energy efficiency by selecting optimal cluster heads in fog supported WSNs. In this protocol, some fog nodes, responsible for sending the gathered data to the internet gateway, must be placed in the sensing area. The protocol considers two-level heterogeneity based on energy and classifies the sensor nodes (which are pre-placed or randomly deployed) into two categories: advanced node and normal node. However, in most scenarios, sensor nodes are placed in a remote area, which limits its deployment in a practical scenario due to its pre-placement requirement. Table 1 gives an overall comparison of existing hierarchical protocols that consider energy hole situation in their literature.

## 3. Proposed WEMER Scheme

Due to the multi-hop communication nature in WSNs, nodes that are closer to the Base Station (BS) need to forward many more data packets than other nodes, and they tend to die much faster because of this many-to-one communication characteristics. As a result, energy holes are created in the inner circles of the BS, and critical data from the relevant regions are not forwarded to the BS, despite having high overall residual energy. As a result, network lifetime decreases very drastically and a significant amount of energy is wasted. An efficient scheme to balance the energy consumption among nodes is therefore inevitable. A layered-based routing technique with a dynamic wedge merging mechanism is proposed here to distribute the energy consumption uniformly across the network. At first, the whole network is divided into unequal width coronas with equiangular wedges, that creates many sectors. A sector is an area between the corona and the wedge. The sensor nodes at each sector then form a chain to transmit data packets toward the sink via Head Node (HN), and HN forwards the data of its sector and are selected based on their remaining energy and distance with its relevant successor HN.

In this paper, we assume that nodes and BS are static in nature, and no mobile sensor node or mobile BS does exist at any time. Furthermore, link asymmetry [46,47] is not considered in this research work. The communication links are assumed to be symmetric in such that two neighbor nodes can always communicate with each other by using the same transmission power. Nodes are required to send their sensed data constantly at a certain rate. For simplicity, we assume that each sensor node generates and sends one packet per round.

### 3.1. Energy Model

The three basic units of sensor nodes are: the sensing unit, the processing unit, and the transceivers. The first order radio model is adopted for the proposed scheme [48]. In this radio model, the electronics energy $E_{elec}$ is used, in order to operate the transmitter or receiver circuit. The transmitter amplifier utilizes both channel models of the free space with $d^{2}$ power loss, and multi-path fading with $d^{4}$ power loss. The radios have power control capabilities and, to reach the intended recipients, can expend the minimum required energy. In order to avoid receiving an unintended transmission, the radios can be turned off. Thus, to transmit a *K*-bit message a distance d using this radio model, the radio expends:

If $d<d_{0}$ than $E_{tr}\left(K,d\right)$ will be

$$E_{tr}\left(K,d\right)=K\times E_{elec}+k\times \epsilon_{fs}\times d^{2}$$

If $d\geq d_{0}$ than $E_{tr}\left(K,d\right)$ will be

$$E_{tr}\left(K,d\right)=K\times E_{elec}+k\times \epsilon_{mp}\times d^{4}$$

And to receive this message, the radio expends:

$$E_{rx}\left(K\right)=K\times E_{elec}$$

where $E_{elec}$ is the unit energy dissipation for transmitter electronics or receiver electronics. $\epsilon_{fs}$ is the amplifier energy in the free space model, while $\epsilon_{mp}$ is the one in the multi-path model, and $d_{0}$ is the threshold and defined as:

(1) $$d_{0}=\sqrt{\epsilon_{fs}/\epsilon_{mp}}$$

### 3.2. Proposed Wedge Merging Scheme

The lifetime of a sensor node mainly depends on the number of alive nodes and connectivity of the network. Once a sensor node is out of energy, it dies prematurely, which affects the performance of the network. The proposed scheme aims to eliminate the energy hole problem in WSN, as well as to maximize the network lifetime. In this section, the WEMER technique is presented in detail. WEMER is divided into the following four phases:

1. Initial setup phase
2. Data transmission phase
3. Merging procedure of a wedge
4. Avoid long link communication

All of the above mentioned phases are discussed in the subsequent sub-sections.

#### 3.2.1. Initial Setup Phase

At the initial setup, the BS divides the whole network into some sector. A sector is the small portion of the network, between the corona and the wedge. We adapt two algorithms described in [49] in order to create coronas and wedges in our proposed scheme.

##### Corona Creation Procedure

**Figure 2:**
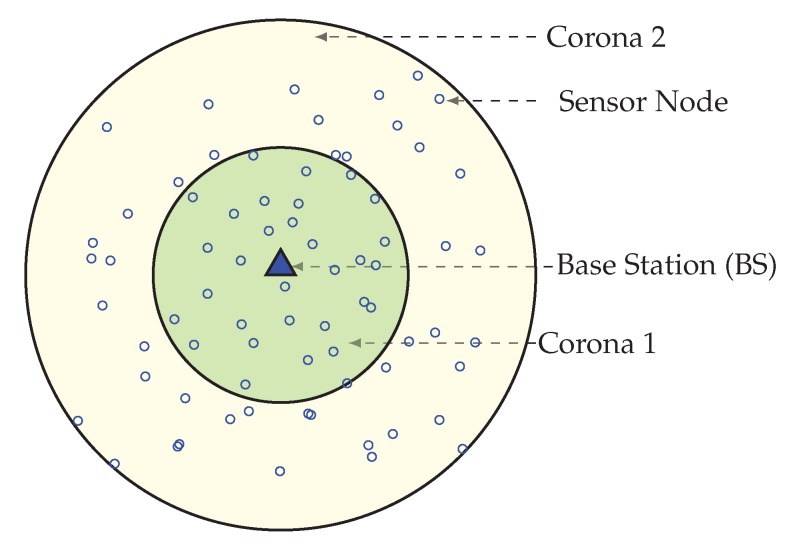
Network divided in 2 coronas.

This section describes how the network area is divided into unequal width, concentric coronas. Before the data transmission starts, each node needs to find its corona. In the network initialization phase, by adjusting transmission power, the BS divides the network into some circularly shaped coronas. The range of transmission for corona creation mainly set by the BS depends on both the requirement of the number of coronas and the node density in the network. The BS sets itself as corona number zero (CN = 0) and adjusts its transmission power equivalent to half of its range. In order to assign a node to coronas BS broadcast, a corona_creation packet must increase its corona number by 1. When a node receives a corona message with CN = i, it sets its corona number to i, if it already not joined to a lower or equal corona. Until all nodes get their corona number from the BS, this corona creation method continues by increasing both the transmission range and the corona number by 1. After these processes, the entire network is alienated into several coronas, that look like concentric circles with center Sink. Figure 2 is showing a network with 2 concentric coronas. The innermost circular region is corona 1 and outermost is corona 2, this is because with the help of the BS, the whole network is divided into two coronas.

##### Wedge Creation Procedure

**Figure 3:**
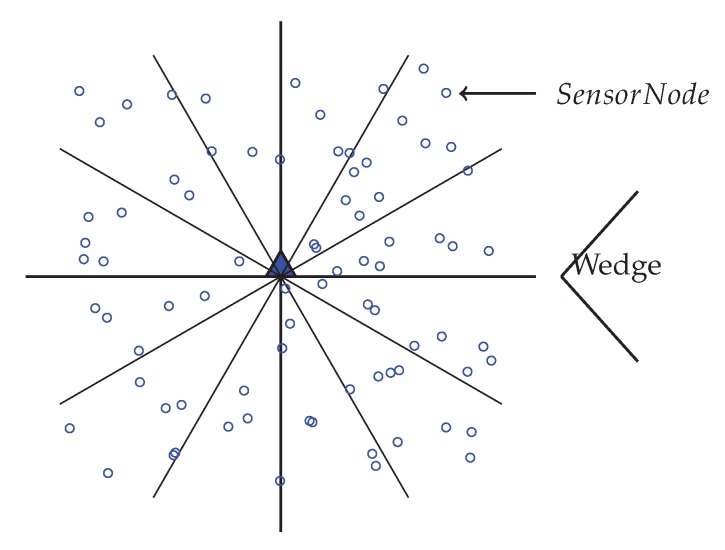
Network with 12 equiangular Wedges.

In order to partition the network into equiangular wedges, the BS directs its antenna to one portion of the network and transmits a wedge_creation packet that contains sink ID and a wedge number. This packet is send out with its maximum transmission power level, to ensure coverage of every node in that direction. When a node receives a wedge_creation packet with a wedge number, then it assigns its wedge to that wedge number, unless it has already joined an equal or lower wedge. Until all nodes get its wedge number from the BS, this wedge creation method continues by both changing the angle of directionality and wedge number by 1. Figure 3 is showing a network with wedges. Here, the entire network gets divided into 12 wedges.

**Figure 4:**
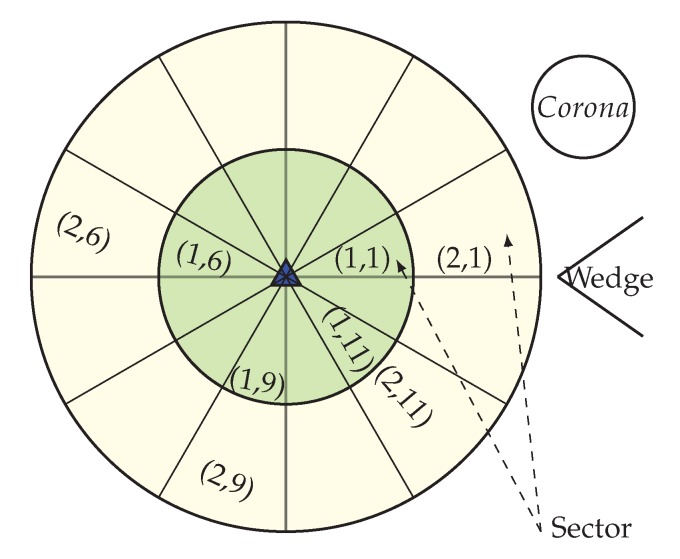
Network with corona, wedge and sector.

After the configuration of the whole network into coronas and wedges, each sensor node gets its corona and wedge number. The corona and wedge creation procedure makes sure that each node belongs to only one sector in the coordinate system. The positions of all the sensor nodes that have the same coordinates form a sector, like a hard disk. A unique sector is defined by the intersection of a wedge and a corona; every sector is identifiable by the combination of a unique corona and wedge identifier. Figure 4 is showing a network with a corona, wedge and sector. In the figure, the corona and wedge number of a node is showing in brackets. Position of corona 1 and wedge 1 for a node is indicated by (1, 1), at the same way corona 2 and wedge 1 are indicated by (2, 1). After the formation every sector, there must be more than one node; if not, the BS then merge that sector’s wedge with its neighbor wedge. According to the distance from the BS, sector size varies. A sector which is closer to the BS is smaller in size, which means there is less intra sector traffic and energy cost. So, the smaller sector consumes less energy and time for the intra sector communication, as they need to use small transmission energy and can concentrate it onto the inter sector communication.

#### 3.2.2. Data Transmission

**Figure 5:**
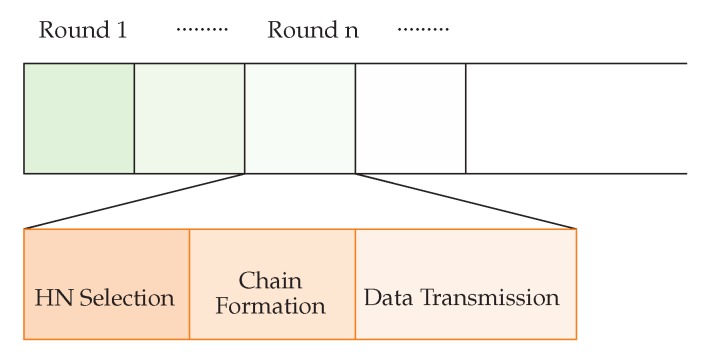
Data transmission Phase.

If only some of the nodes in the network participate with all of the processes, while others are inactive or idle, then it produces a greater chances for that node to die early and network partitioning eventually decreases the network lifetime. WEMER works in rounds and according to Figure 5 every round is further divided into three phases.

In order to send the sensing information to the BS, every wedge constructs a virtual path which will join the outermost sector to the BS. A node then selected as a Head Node from a sector, and that Head Node sends the cumulative traffic of its sectors, and its predecessors to its successors. Only one node from a sector is used to forward the data to the BS, so MAC level contention is greatly reduced.

##### Head Node Selection Phase

In WEMER, the position of the BS is in the center of the network, and the HN selection process starts from the inner most sector of the network. There is only one HN for every sector. A HN can be selected for a sector by both the node’s RE and the distance with its relevant successor HN. A node which has less RE than its sector’s average energy can not participate in the HN selection competition. In the HN selection phase, nodes from a sector sets a timer ($T_{i}$) according to the following equation, in order to become a HN,

(2) $$T_{i}=\frac{Total\_adv\_time}{E_{i}+\frac{d_{0}}{D_{itoBS}}}$$

(3) $$T_{i}=\frac{Total\_adv\_time}{E_{i}+\frac{d_{0}}{D_{itoHN}}}$$

**Figure 6:**
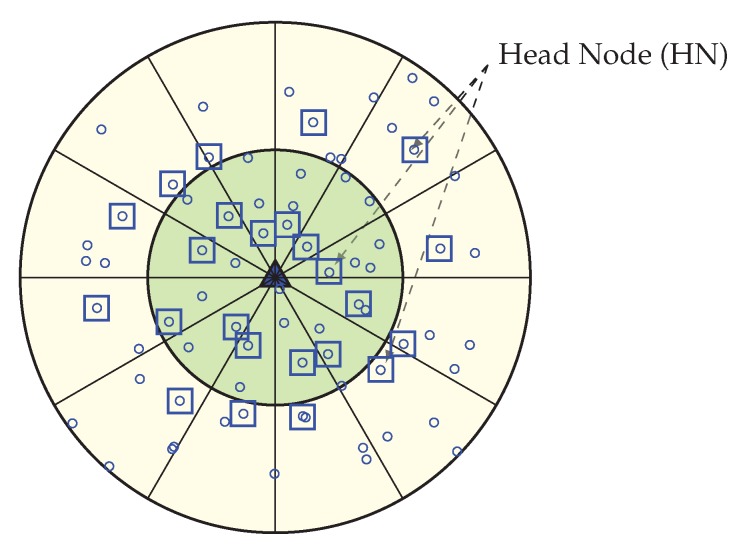
Selection of a Head Node (HN) for each sector.

Here $E_{i}$ is the RE of the node, $d_{0}$ is the threshold distance obtained from Equation 1, $D_{itoHN}$ is the distance between the node *i* and its successor head node (in case of CN=1), $D_{itoBS}$ is the distance between the node *i* and the BS (in case of CN>1). The HN selection procedure starts from the corona 1. Here, every node set a timer ($T_{i}$) where Total_adv_time is divided by the summation of RE ($E_{i}$) and distance factor ($d_{0}$/$D_{itoBS}$), so that higher residual energy and less distance node can send HN_Msg earlier within the total advertisement time (Total_adv_time). After receiving HN_Msg sensor nodes that are in the same sector, the node leaves the competition and nodes that belongs to the successor sector calculate the distance based on the RSSI of the HN_Msg packet. Node belongs to Corona 2 sets their timer according to Equation 3 and in the same procedure node which has high residual energy and less distance between the predecessor HN sends HN_Msg packet. Figure 6 is showing the Head Node for every sector. This HN selection process run at every round in order to select the most capable node as HN, and until any node becomes more capable, the same HN can operate for many continuous rounds. If any node becomes better than the current HN of its sector again, a new HN selection phase is called and the most suitable node become new HN. The pseudo-code of the head node selection procedure is shown in Algorithm 1.
**Algorithm 1:** Head node selection procedure for sector
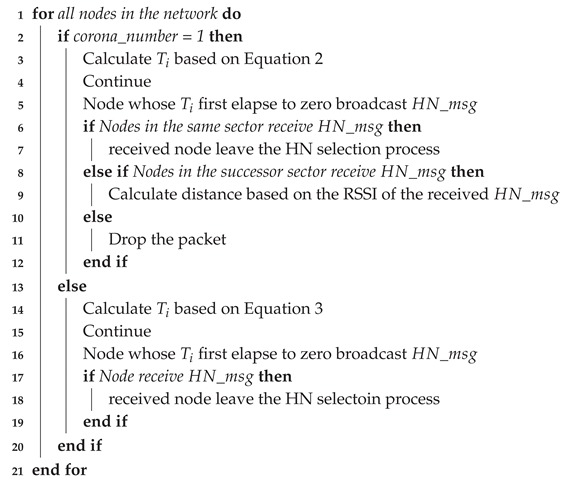


##### Chain Construction Phase

The main concern of the chain construction phase is to find a low cost chain that covers all nodes of the sector. PEGASIS and CCS use greedy algorithms in order to build a chain, starting from the farthest node of the network. A greedy algorithm is always a good choice for chain construction, but for large networks it causes a serious problem that leads to the long link(LL) problem [50,51].

**Figure 7:**
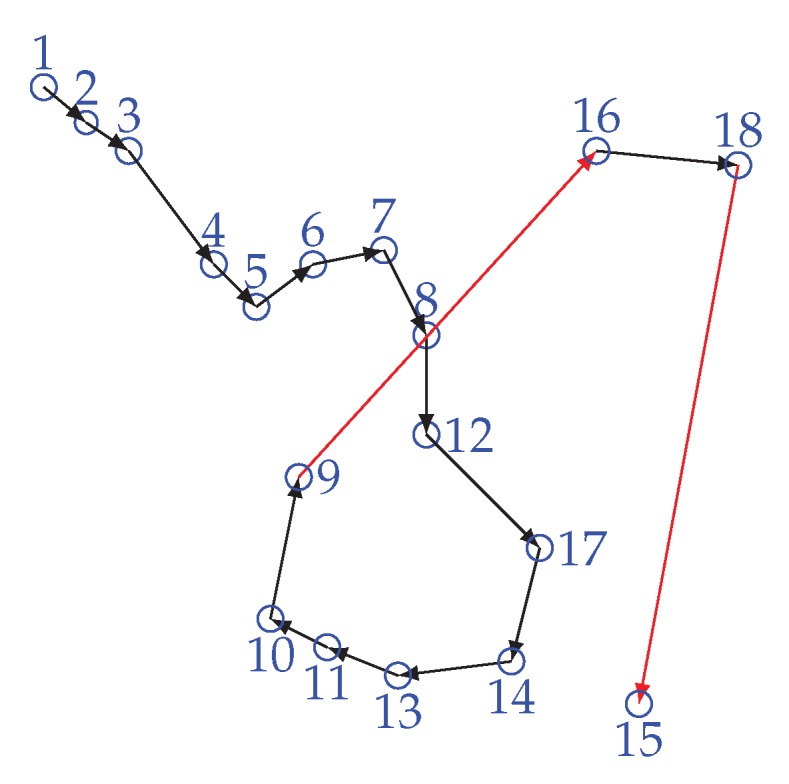
Long link problem.

**Figure 8:**
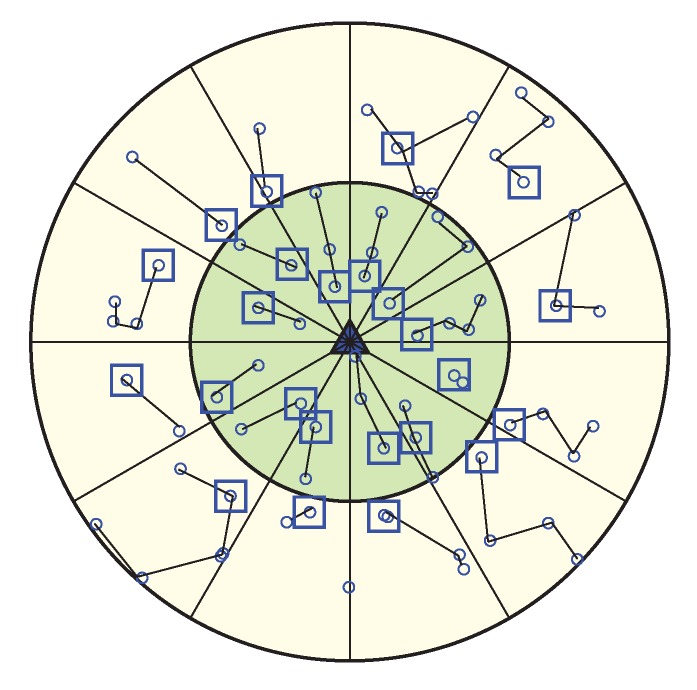
Chain construction for every sector with head node.

Figure 7 is showing the scenario of the LL problem. In order to solve the LL problem, the proposed scheme makes an enhancement over the greedy method. Instead of connecting with the next minimum distance node that is not in the chain, WEMER connects with a node that is already in the chain, and with whom distance is minimum. In each sector, the chain construction procedure will be started from the farthest node from the head node and it joins the chain first. Then, it finds the distance between itself and other nodes which have not joined the chain yet. It finds the nearest node and sets it as node *i* waiting to join the chain, *i* represents the $i^{th}$ node joined. Node *i* gets the information of distance between itself and i−1 nodes, which are on the chain, it then finds the nearest node *j* and directly connects with it to join the chain. At this point node i becomes the new end node of the chain. This procedure continues until all sensor nodes have joined the chain, so that a branching chain is finally formed. Figure 8 is showing the chain between node for every sector.

In order to distribute the energy load evenly among all nodes, LL should be avoided. This new chain creation procedure solves the LL(Long Link) problem of greedy algorithms. Thus, a new chain construction method ensure less energy consumption of nodes. The pseudo-code of chain construction procedure is shown in Algorithm 2.
**Algorithm 2:** Chain construction procedure for each sector
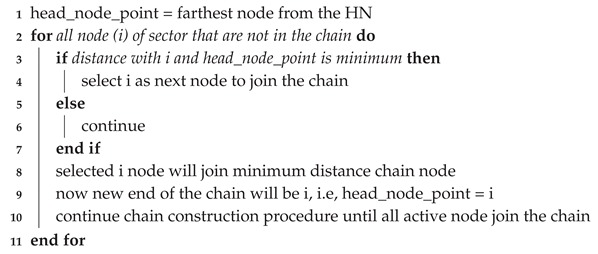


##### Data Communication Phase

**Figure 9:**
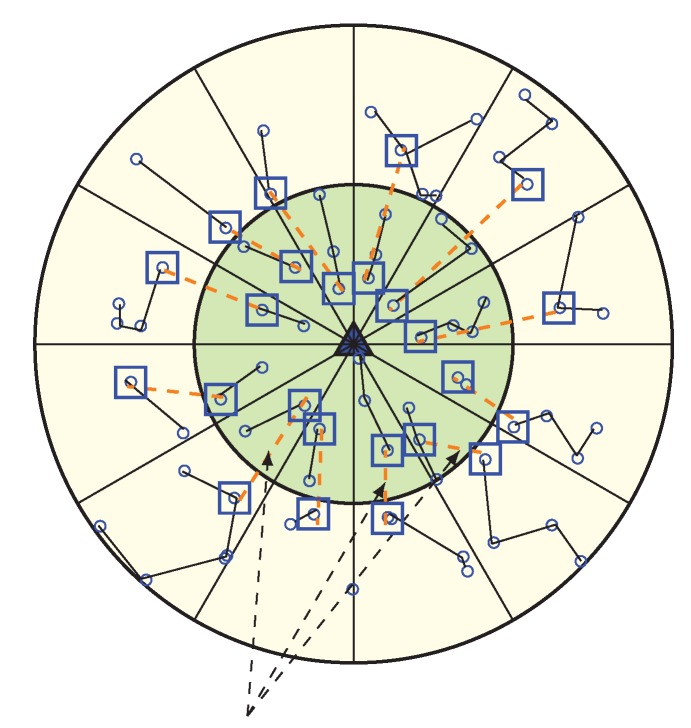
Data Communication Phase.

After WEMER performs the first two phases, the HN initiates a token passing approach in order to start the data transmission from the maximum distance node to the HN of the chain. The chain formed in WEMER has more than two end nodes. If, for a node, the connected node count is zero; i.e no node is connected with it, the node is called as the end node/tail node of that chain. As each chain may have more than two end nodes, before data transmission, every end node needs to inform whether it is a head node or not to its connected node. This information helps the flow of data transmission. As a chain has more than two end nodes, it is necessary to know the right path to reach the HN. For this reason in token passing approach every end node informs its connected node if it is HN or not. The pseudo-code of the data communication procedure is shown in Algorithm 3. All nodes in each sector transmit their data along the chain to its selected node, in its slotted time. In order to deliver data, every node send its sense data to its selected neighbor node, in its time slots, assigned by the TDMA mechanism, and after receiving the data, the neighbor node fuses them with its own data and forwards these to its selected neighbor node or HN. After collecting all the data from sector, HN sends this data to its or HN. When the HN within Corona 1 of a sector receives all the data from its predecessor sector, after aggregation, sent all these data to the BS. As the data transmission procedure starts from the outer most coronas, for these reason when Sink receives data from the HN of its corona 1, then one round is complete. In this scheme, the HN in every sector is changed in every round according to distance and energy, thus it reduces the burden of some nodes to elect repeatedly for the HN. Figure 9 is showing connection between nodes along with links between the HN.
**Algorithm 3:** Data Communication procedure
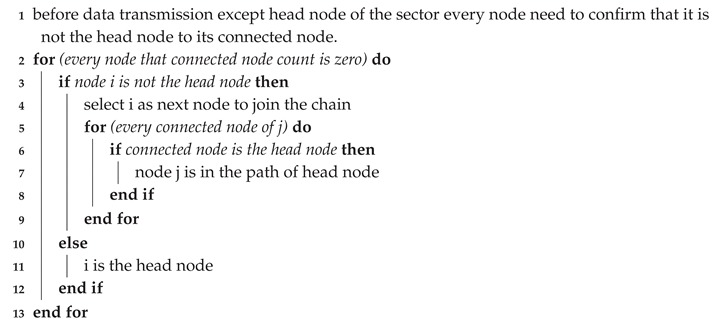


#### 3.2.3. Merging Procedure of a Wedge

The result of the energy hole is a network partition along with sink isolation and network failure. Moreover it degrades network performance. For this reason the energy hole problem needs to be eliminated in order to maximize the network lifetime. So after the data transmission phase of every round, WEMER checks the average energy of every sector. This is because, if any sectors average energy become low, it creates an energy hole in that portion of the network. For this cause, the wedge which has a sector with low energy then merge with one of the neighboring wedges.

##### Wedge Merging Step

**Figure 10:**
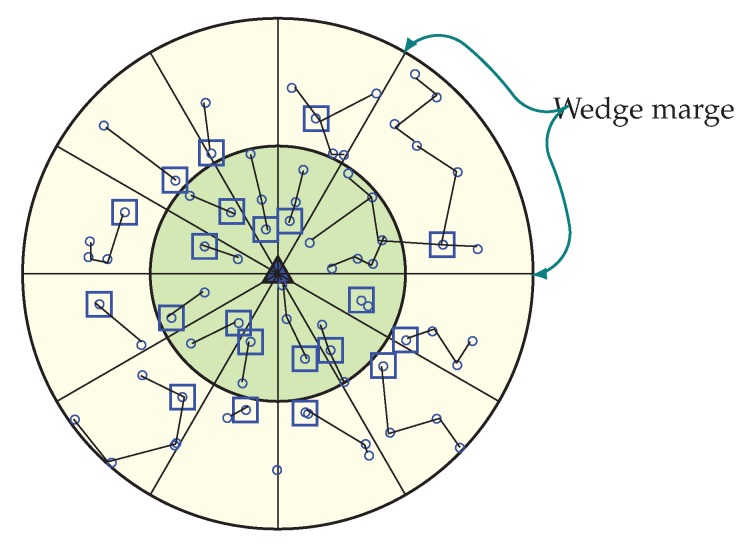
Occurrence of first wedge merge with a new chain formation and Header Node (HN) selection.

In our WEMER scheme, wedge merging happen for two reasons. First, in the network initialization phase after the formation of both the corona and wedge, BS check if every sector has more than one node; if not, BS will then merge the wedge of that sector with its neighboring wedge. Second, in the data transmission period, sensor nodes lose their energy to transmit and receive data. So, every head node needs to check for the average energy of its sector after every round. When the RE of a sector becomes less than 40% of its initial energy, the corresponding wedge, belonging to the sector with reduced RE, get merge with one of the neighboring wedges. Figure 10 is the scenario of network after the first wedge merge.

Afterwards, every round, a HN belonging to each sector checks whether the average energy of the sector reaches 40% of its initial energy or not. If it becomes less, the HN starts the wedge merging procedure. In order to start merging wedges, the HN of the lower RE sector sends a beacon named avg_en to its two neighboring wedges, in order to inform them of their average energy. When any HN receives an avg_en beacon from a HN, it responds by sending its sector’s average energy to that HN, as every HN is acquainted with its own sector’s average energy. After the response from the neighboring wedge’s head node, the wedge with the higher energy head node is selected as a wedge to merge. The wedge merging procedure starts from the inner most sector of the corresponding wedge that contains the low energy sector, and ends at the outermost sector of that wedge. Suppose that a sector with the position (2, 2) has a low energy and decides to start merging, it will then send avg_en beacon to the sectors with position (2, 3) and (2, 1) by sending their average energy. After receiving avg_en, the beacon head node responds by sending their average energy, and sector (2, 3) will be selected for merging, as this sector has more average energy than sector (2, 1). At first, sector (1, 3) and (1, 2) get merge and all nodes from sector (1, 2) updates their sectors as (1, 3). After that, sector (2, 2) and (2, 3) are merge, and this procedure continues until the outermost sector merges.

##### Update of Nodes Position

**Figure 11:**
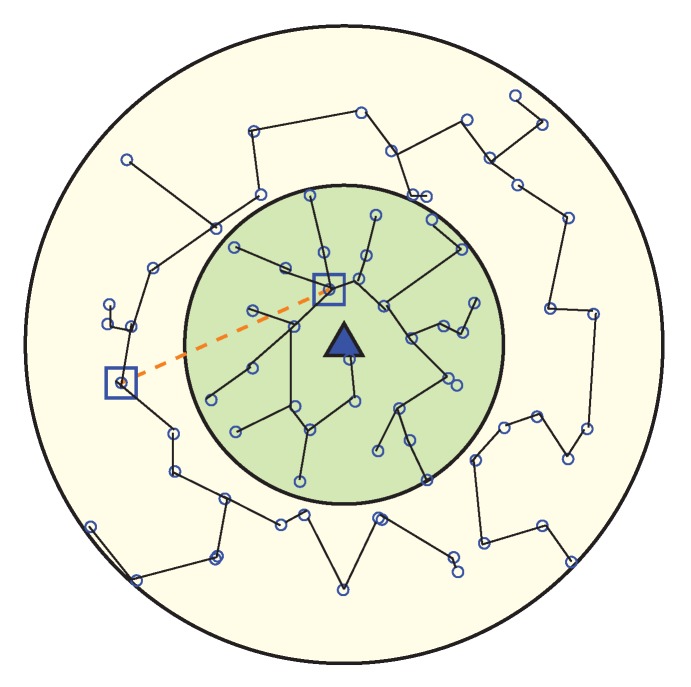
Network topology after all wedges are merged.

In the wedge merging procedure, every node in the merge wedge updates their position according to the new wedge; in this way two different wedges become one. Additionally, neighbors of merged wedges update their neighbor’s information. The new wedge selects its head node and then creates new chain for data communication. In Figure 10, the first merged wedge is showing. After completing several round in the network, every sector has enough energy in order to continue its data transmission but, the HN of sector (2, 2) found that its average energy is lower than 40% of its initial energy, so it starts to merge with another sector. From two neighbor sectors (2, 1) and (2, 3), it found that sector (2, 1) has more energy than sector (2, 3). Thus, it merge with sector (2, 1) and a new sector is constructed, shown in Figure 10. As the low energy sector is merged with the sector of high energy, it again gets more high energy nodes to make a chain. Figure 11 is showing when all wedges merge in the network.

#### 3.2.4. Avoid Long Link Communication

In order to make the WEMER efficient, the distance threshold concept is used. When one or more wedges merge, a node that is farthest from the HN needs to travel a long distance in order to send their data to the HN. This introduces a longer link in data transmission. In order to avoid this long link, a new concept of using the distance threshold ($d_{th}$) is proposed in order to reduce data from long link traverse of the network. In a sector, when the maximum distance between two nodes become greater than the $d_{th}$ value, more than one HN can be created for that sector.

**Figure 12:**
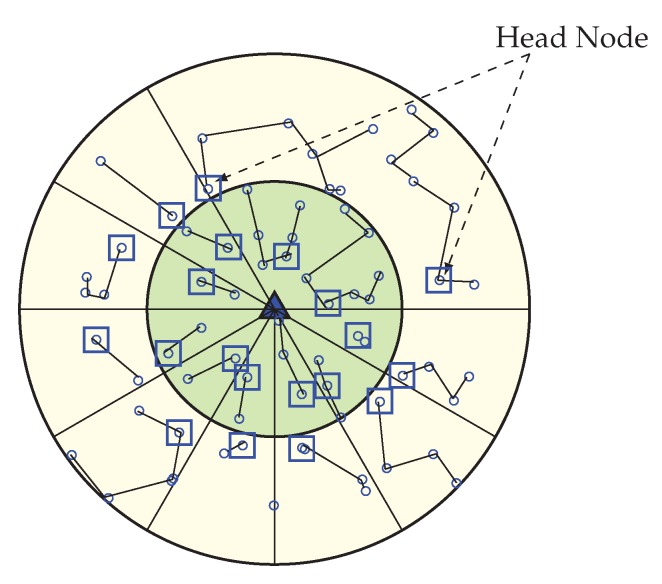
Avoidance of long distance communication by selecting more than one HN for a sector.

After wedge merging, every node calculates their distance with the other nodes of the sector. If the maximum distance between two nodes is greater than $d_{th}$, then, according to the distance, more HNs can be selected for that sector. As width of corona one and corona two is not same, two different distance thresholds are used. For corona one, the distance threshold is 30, whereas for corona two, the distance threshold is 45. Figure 12 is showing two HNs in a sector.

**Figure 13:**
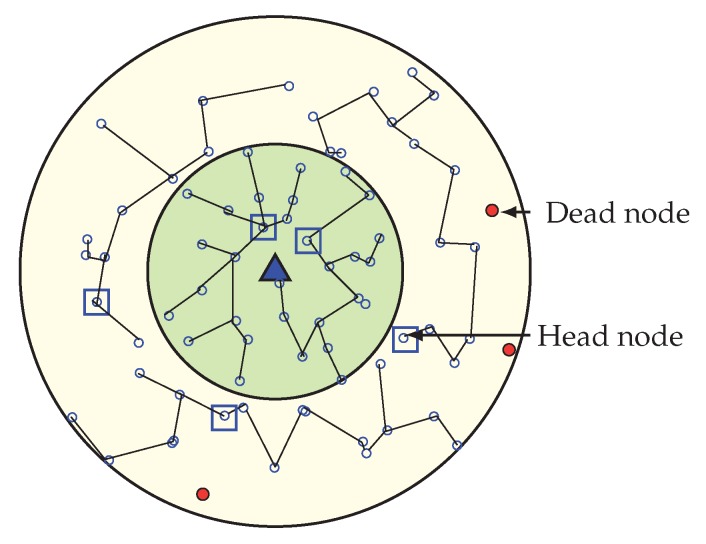
When all wedges merge.

Multiple HN selection procedure is similar as single HN selection type, however, the difference is that, in order to make a certain distance between the HN when the first HN selects, it sends HN_Msg to its sector within transmission range $d_{th}$. The nodes who receive this message can not participate for any other HN selection competition during this round, and the node who did not receive any message continues the head node selection procedure in order to be the second HN for a sector. This procedure continues until all nodes receive HN_Msg. Figure 13 is showing the scenario of HN when all wedges merge in the network. After the HN selection procedure, in order to construct a chain, nodes join to the HN which is closest to it. The chain construction and data transmission technique is the same as a single HN procedure type.

## 4. Simulation and Result

In this section, extensive simulation results are provided, with comparisons of performance between the proposed WEMER technique and three other popular methods: Power Efficient GAthering in Sensor Information Systems (PEGASIS), Concentric Clustering Scheme (CCS), and Multilayer Cluster Designing Algorithm (MCDA). This is done to study the performance of energy consumption, the network lifetime, as well as alive and dead nodes over rounds in this section.

Simulation is done in the MATLAB [52] environment in order to evaluate the performance of the proposed WEMER scheme with the three popular protocols PEGASIS, CCS and MCDA. By using the simulation result with different simulation scenarios, WEMER is compared with the previous protocol.

### 4.1. Experiment Setup

Table 2 shows the parameters used in the simulation. A homogeneous sensor network is assumed, in which all sensor nodes have equal sensing and processing capabilities initially. Each node has the initial energy of 0.5 Joule, and the length of the data message is 4000 bits. Our selection of these values is highly motivated by [45,53,54,55,56]. Each sensor node has location information, and no mobility is taken in account to mean that nodes are static in nature. In the proposed scheme, the data is passed through a multi-hop approach.

### 4.2. Results and Discussion

In this section, the performance analysis of the WEMER scheme is carried out through simulation results. Performance improvement in terms of network lifetime, energy cost, and balanced energy consumption is investigated and compared to existing protocols: PEGASIS, CCS and MCDA.

#### 4.2.1. Residual Energy of Network per Round

**Figure 14:**
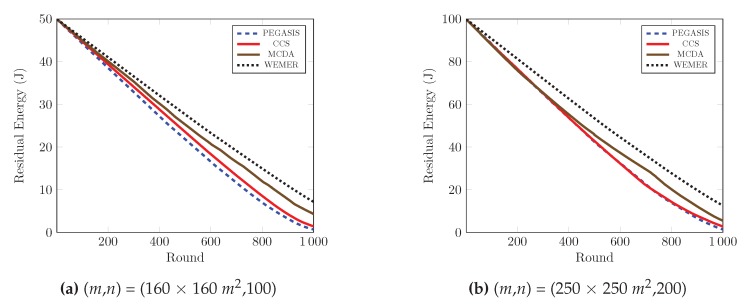
Residual energy of network with respect to round

The remaining energy of the sensor nodes after each round, affects the network lifetime directly. If the remaining energy of the nodes is high, then the network lifetime is high; and vice versa. Figure 14 demonstrates the performance comparison in terms of residual energy of the network, with respect to the round. Figure 14a shows that, at round 600, the remaining energy of the network is 33.1%, 36.65% and 41.45% for PEGASIS, CCS and MCDA respectively, while in the proposed protocol, the remaining energy of the network is 46.56% of the initial energy. Furthermore, at round 1000, the remaining energy of the proposed protocol is greater than 6.47 J, 5.7 J, and 2.87 J compared with the PEGASIS, CCS and MCDA protocols respectively. It is clear from the figure that proposed protocol is more energy efficient than PEGASIS, CCS and MCDA, as the selection of the HN for every sector depends on both the distance with the relevant HN and residual energy, the nodes that contain low residual energy and are isolated from other nodes of the sector, can not be selected as Head Node. Figure 14b demonstrates the results of the same metric for 250 × 250 m${}^{2}$ area with 200 nodes, and it also agrees with the previous characteristics.

#### 4.2.2. Number of Alive Nodes over Round

**Figure 15:**
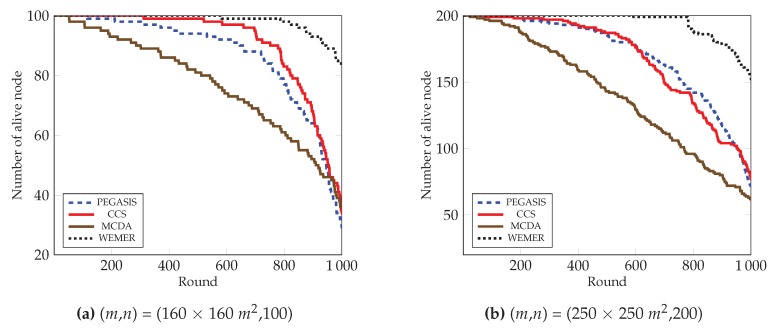
Number of alive nodes over round.

To prolong the network lifetime of network, the number of alive nodes is an important parameter. Figure 15 clearly depicts that the number of alive nodes in the network is higher than the competitive protocols. At round 800 in Figure 15a, 98% of the nodes are still alive in the proposed protocol, whereas 77%, 83%, and 61% of the nodes are alive in the network for the PEGASIS, CCS, and MCDA protocols respectively. Accordingly, at round 1000, 56%, 50% and 48% more nodes are alive in the proposed protocol, as compared to the existing PEGASIS, CCS, and MCDA protocols respectively. This is because the PEGASIS protocol constructs a chain by using a greedy method, thus it creates a Long Link (LL) between nodes during chain construction, so that nodes deplete energy very fast and die early. CCS also uses a greedy method in order to construct a chain and also suffer from LL, whereas MCDA is free from LL, however, because its layer 1 nodes directly communicate with the BS, the nodes in the boundary deplete their energy much faster than any other node. In contrast, the chain construction procedure of proposed WEMER is so efficient that it reduces both the distance and LL between sensor nodes. Additionally, HN selection depends on the distance between the or HN or BS and the energy, so all nodes get the chance to be a HN. All of these considered parameters of WEMER reduce energy consumption of nodes and increase the lifetime of the network. Figure 15b also shows that the proposed protocol significantly outperforms PEGASIS, CCS, and MCDA protocols.

#### 4.2.3. Number of Dead Nodes over a Round

**Figure 16:**
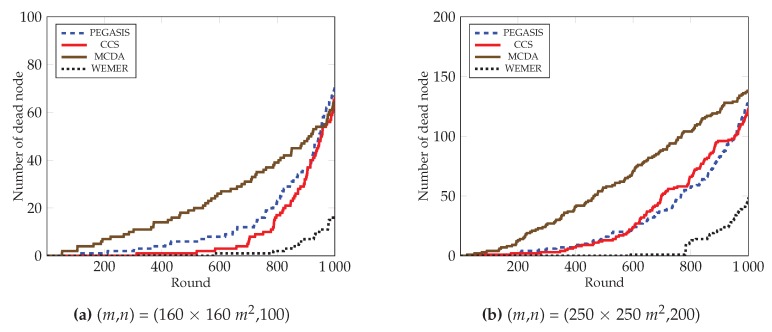
Number of dead nodes over round.

In Figure 16, the number of dead node over the round of all protocols is demonstrated. It is clear from the figure that the number of dead nodes in the network of the proposed protocol is much less than the existing protocols. The proposed protocol deals with the load balance by creating sectors, and then selecting the most fit node as head node. As a result, the proposed protocol prohibits the sudden death of the node. At round 700, only 1% of nodes have died in the proposed protocol. In contrast, 12%, 5%, and 31% nodes die in the PEGASIS, CCS, and MCDA protocols respectively. Both the PEGASIS and the CCS protocols face the long link problem that has already been discussed; in such a situation, nodes need to forward data in a long path. This eventually causes too much energy consumption, as well as the premature death of nodes. From Figure 16b, it is also observed that at round 800, only 6% nodes die in case of the proposed protocol, whereas the number increases to 28.5%, 33%, and 52% for PEGASIS, CCS, and MCDA protocols respectively.

#### 4.2.4. Percentage of Dead Node

**Figure 17:**
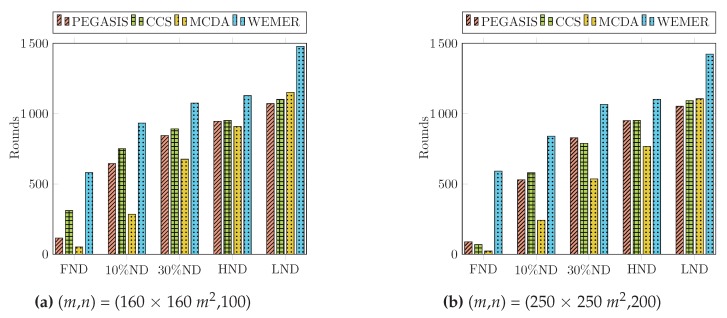
Percentage of dead node.

The condition of the network can easily be identified through First Node Die (FND), Half Node Die (HND), and Last Node Die (LND). In this paper we also carried out two more parameters: 10% Node Die (TND) and 30% Node Die (THND), in order to compare the performance. The instability period of the network starts after the FND occurs, as shown in Table 3. The percentage of dead nodes also demonstrates the total lifetime of the network in terms of a round. Figure 17a clearly shows that for PEGASIS, CCS, and MCDA, the first node dies at round 115, 311, 52, however, for WEMER, the first node dies at round 582. Fifty percent and Last Node Die for WEMER at round 1128 and 1478 respectively, whereas for PEGASIS, CCS, MCDA, fifty percent and Last Node Die at round 945, 951, 909 and 1071, 1102, 1154 respectively. In the case of the MCDA protocol, the FND and TND occur early, but the moderate load balancing scheme extend the lifetime of nodes, as compared to PEGASIS and CCS. From Figure 17b and Table 4, it is also obvious that WEMER outperforms PEGASIS, CCS, and MCDA in terms of network stability period considering 200 nodes and 250 × 250 m${}^{2}$ area.

If all node of corona 1 died and node of corona 2 is still active, than corona 2 node directs communicate with base station. The detailed network condition about how the nodes connect after nodes die are given below:

##### Network After First Node Died

**Figure 18:**
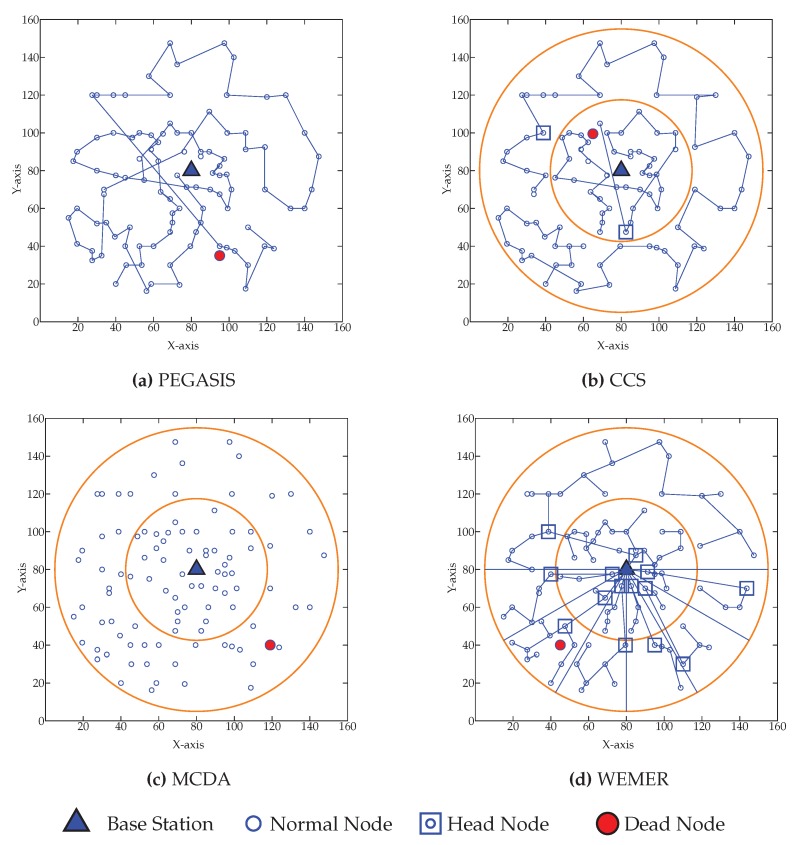
Network topology after First node die (**a**) at 115th round for Power Efficient GAthering in Sensor Information Systems (PEGASIS) (**b**) at 311th round for Concentric Clustering Scheme (CCS) (**c**) at 52th round for Multilayer Cluster Designing Algorithm (MCDA) (**d**) at 582th round for WEMER.

Figure 18 shows the network condition when first node died for WEMER and all three compared protocol. Figure 18a is showing the node’s data transmission condition for PEGASIS. For PEGASIS, at round 115 the first node died and when a node died a new chain is constructed bypassing that dead node. At the same way for CCS, MCDA and WEMER’s first node died at round 311, 388, 582 and are showing in Figure 18b–d. When the node died in the network, that node cannot take part in data transmission, so it is necessary to make energy consumption of every node very low so that it dies after a long time. It is very clear from the figure that WEMER performs better than all three protocols. The more nodes involved in data transmission process, the more it helps BS closer nodes to save their energy or protect high energy consumption. As chain construction procedure and HN selection took place after every round so it make sure that no low residual energy node select as HN and no node select HN repeatedly.

##### Network After Ten Percent of Node Died

**Figure 19:**
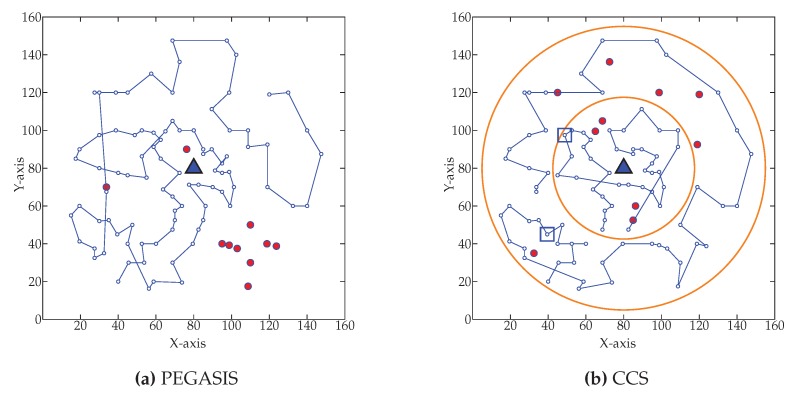

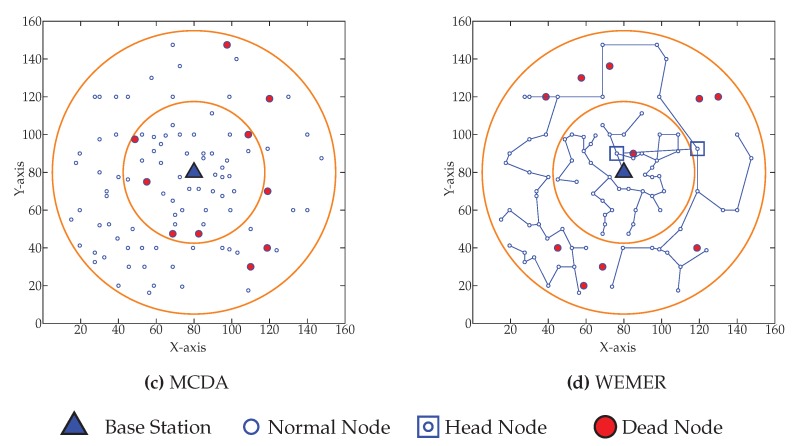
Network topology after Ten percent node die (**a**) at 645th round for PEGASIS (**b**) at 751st round for CCS (**c**) at 601st round for MCDA (**d**) at 938th round for WEMER.

Figure 19 shows the network condition when ten percent node dies for WEMER and three comparative protocols. Figure 19a shows the node’s data transmission condition for PEGASIS. For PEGASIS, ten percent of its node dies at 645th round. Similarly, the same percentage of nodes dies at 751st, 601st, and 933rd round for CCS, MCDA, and WEMER protocols demonstrated in Figure 19b–d respectively.

##### Network After Fifty Percent of Node Died

**Figure 20:**
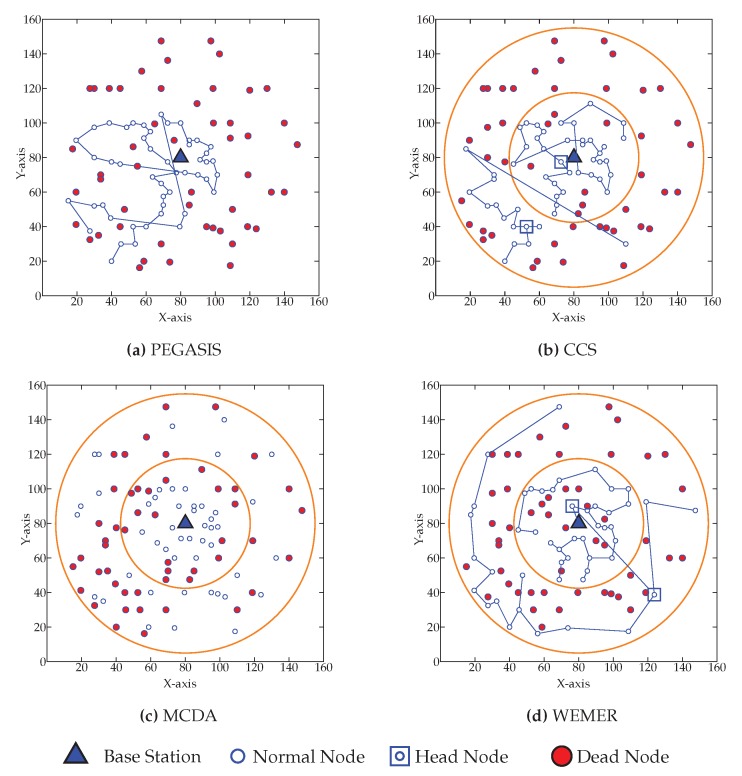
Network topology after Fifty node die (**a**) at 945th round for PEGASIS (**b**) at 951st round for CCS (**c**) at 987th round for MCDA (**d**) at 1128th round for WEMER.

Figure 20 shows the network topology when fifty percent of nodes die. Figure 20a shows that fifty percent node dies at 945th round in PEGASIS. Figure 20b–d show that the same percentage of nodes die for CCS, MCDA and WEMER at 951st, 987th, 1128th round respectively.

#### 4.2.5. Average Energy Cost of a Node for a Round

**Figure 21:**
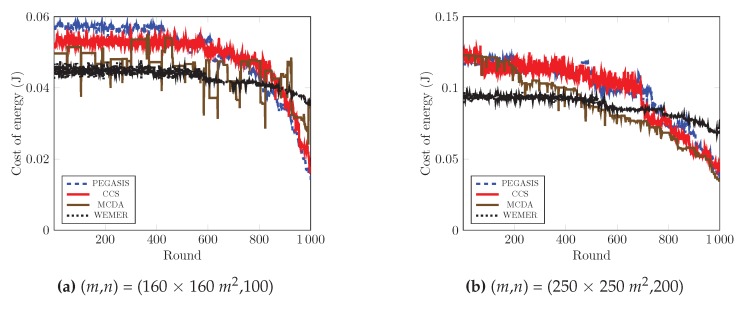
Average energy cost over round.

To maximize the lifetime of the network, the energy cost per round is another essential parameter. Figure 21a clearly shows that at round 800, CCS and PEGASIS consumes 0.05 J per round and then the consumed energy abruptly drops as the energy cost of the network is greatly reduced due to the fact that most of the nodes die beyond 800th round. On the other hand, MCDA’s cost per energy is in a zigzag manner, thus, sometimes the network consumes high energy and sometimes low energy. In contrast, the proposed protocol consumes energy in a stable manner. The proper head node selection and wedge merging scheme allows the network to consumes a stable rate and prohibits the sudden death of the network. As shown in Figure 21a and Table 3, the average energy cost of the network is 0.049297 J, 0.0485402 J, and 0.045712 J for PEGASIS, CCS, and MCDA respectively, whereas it is 0.042847 J for the proposed protocol. Furthermore, the maximum energy consumption in any particular round is 0.044642 J for the proposed protocol, in contrast to 0.058585 J. 0.053361 J, and 0.49652 J for PEGASIS, CCS, and MCDA respectively. Besides, Figure 21b and Table 4 demonstrate that the proposed WEMER reduces the energy cost by 12.70%, 11.14%, and 0.82% as compared to PEGASIS, CCS, and MCDA respectively.

## 5. Conclusions

Extending the network lifetime is one of the most challenging issues in WSNs. Uneven energy consumption among the nodes contributes to the creation of energy holes, which significantly decreases the network lifetime. In this research work, a dynamic WEdge MERging (WEMER) based scheme named WEMER is proposed, that minimizes energy hole creation by dividing the whole network into a number of coronas and wedges, and then merging a wedge with one of the neighboring wedges whenever it is likely to have a hole. In the head node selection phase, WEMER considers both the residual energy of each node, and the distance between node and successor HN as parameters to select the most efficient head node. This makes the energy consumption among nodes more balanced. For routing, WEMER uses an enhanced version of a greedy algorithm in order to construct the chain between nodes, which effectively makes the energy consumption lower. Simulation result shows that the proposed WEMER scheme outperforms the contemporary schemes in terms of network lifetime, energy consumption, and energy cost.

## Figures and Tables

**Table 1:** Comparison of existing protocols mitigating energy hole problem.

Existing Protocol	Hierarchical Method	Load Balancing	Selection of CH	Communication Mechanism	Recovery In Case of Energy Hole
PEGASIS [35]	Chain	Low	Probabilistic	Single Hop	No
CBCCP [36]	Chain	Low	Probabilistic	Single Hop	No
CCS [37]	Chain	Moderate	Probabilistic	Single Hop	No
MCDA [38]	Chain	Moderate	Probabilistic	Single Hop	No
EEEHR [39]	Cluster	Moderate	Probabilistic	Multi Hop	No
EEUCLC [40]	Cluster	High	Deterministic	Multi Hop	No
Elkamel et al. [41]	Cluster	High	Deterministic	Multi Hop	No
Wang et al. [42]	Layer	High	Deterministic	Multi Hop	No
ICSPC [43]	Layer	High	Deterministic	Multi Hop	No

**Table 2:** System Parameters

Parameters	Value
BS location	(80m, 80m)
Area, *m*	(160 × 160 m^2^), (250 × 250 m${}^{2}$)
Number of Nodes, *n*	100, 200
Initial Energy	0.5 Joule
Packet Size	4000 bits
$E_{elec}$	50 nJ/bit
$\epsilon_{fs}$	10 pJ/bit/m${}^{2}$
$\epsilon_{mp}$	0.0013 pJ/bit/m${}^{4}$
$E_{DA}$	5 nJ/bit/signal

**Table 3:** Comparison of the proposed WEdge MERging (WEMER) with contemporary protocols in terms of longevity and energy consumption when area is 160 × 160 m${}^{2}$ and N = 100.

Protocol	Stability Period	Instability Period	Network Lifetime Round	Average Energy Cost
PEGASIS	1–115	116–1071	1–1071	0.049297 J
CCS	1–311	312–1154	1–1154	0.0485402 J
MCDA	1–388	389–1163	1–1163	0.045712 J
WEMER	1–582	583–1478	1–1478	0.042847 J

**Table 4:** Comparison of the proposed WEMER with contemporary protocols in terms of longevity and energy consumption when area is 250 × 250 m${}^{2}$ and N = 200.

Protocol	Stability Period	Instability Period	Network Lifetime Round	Average Energy Cost
PEGASIS	1–89	90–1052	1–1052	0.098563 J
CCS	1–69	70–1093	1–1093	0.097196 J
MCDA	1–23	24–1106	1–1106	0.08812 J
WEMER	1–591	592–1423	1–1423	0.087452 J

## Abbreviations

The following abbreviations are used in this manuscript:

HN	Head Node
WEMER	WEdge MERging
PEGASIS	Power Efficient Gathering in Sensor Information Systems
CCS	Concentric Clustering Scheme
MCDA	Multilayer Cluster Designing Algorithm
WSNs	Wireless Sensor Networks
BS	Base Station
RE	Residual Energy
EHP	Energy Hole Problem
ADRP	Adaptive Decentralized Re-clustering Protocol
CH	Cluster Head
MDC	mobile data collector
CCO	Cluster Coordinator
DCD	Distributed Cluster Designing
CCD	Centralized Cluster Designing
CCS	Concentric Clustering Scheme
CN	Corona Number
WN	Wedge Number
LL	Long link
TDMA	Time Division Multiple Access
FND	First Node Die
HND	Half Node Die
LND	Last Node Die
TND	10% Node Die
THND	30% Node Die
